# Update on the Brassicaceae species checklist

**DOI:** 10.3897/BDJ.9.e58773

**Published:** 2021-03-04

**Authors:** Ardath Francis, Beatriz E Lujan-Toro, Suzanne I Warwick, James A Macklin, Sara L Martin

**Affiliations:** 1 Agriculture and Agri-Food Canada, Ottawa, Canada Agriculture and Agri-Food Canada Ottawa Canada

**Keywords:** checklist, Brassicaceae, mustard family, crucifer, global checklist

## Abstract

**Background:**

Here we present a revised species checklist for the Brassicaceae, updated from Warwick SI, Francis, A, Al-Shehbaz IA (2006), Brassicaceae: Species checklist and database on CD-ROM, Plant Systematics and Evolution 259: 249─25. This update of the checklist was initiated, based on recent taxonomic and molecular studies on the Brassicaceae that have resulted in new species names, combinations and associated synonyms.

**New information:**

New data have been added indicating tribal affiliations within the family and where type specimens have been designated. In addition, information from many early publications has been checked and added to the database. The database now includes information on 14983 taxa, 4636 of which are currently accepted and divided into 340 genera and 52 tribes. A selected bibliography of recent publications on the Brassicaceae is included.

## Introduction

The taxonomic organisation of the Brassicaceae has continued to undergo revision and refinement. Extensive taxonomic and molecular studies from many parts of the world have led to several additional taxa, nomenclatural changes in existing taxa and changes in tribal affiliations within the family. Verification of many previously unavailable references has been made possible due to recently digitised archival material dating from the 18th to the early 20th centuries. Additionally, the publication of the results of extensive surveys of the Linnaean and other herbaria has led to the typification of specimens in cases where an author’s holotype was not clear. This information has been incorporated into the species checklist.

The current checklist links all listed taxa to 52 tribes, including several that have been recently described or re-established: Alyssopsideae (Warwick et al. 2010), Aphragmeae (German and Al-Shehbaz 2008), Asteae (Warwick et al. 2010), Biscutelleae (German and Al-Shehbaz 2008), Bivonaeeae (Koch 2012), Calepineae (German and Al-Shehbaz 2008), Conringieae (German and Al-Shehbaz 2008), Crucihimalayeae (German and Al-Shehbaz 2010), Dontostemoneae (Al-Shehbaz and Warwick 2007), Erysimeae (German and Al-Shehbaz 2008), Eudemeae (Warwick et al. 2010), Hilliellieae (Chen et al. 2016), Kernereae (Warwick et al. 2010), Malcolmieae (Al-Shehbaz and Warwick 2007), Megacarpaeeae (German 2010), Microlepidieae (Warwick et al. 2010), Notothlaspideae (Warwick et al. 2010), Oreophytoneae (Warwick et al. 2010), Scoliaxoneae (Warwick et al. 2011), Shehbazieae (German and Friesen 2014), Stevenieae (Al-Shehbaz et al. 2011b) and Yinshanieae (Warwick et al. 2010). The originally broadly-based tribe Malcolmieae (Al-Shehbaz and Warwick 2007) now includes only the genus *Malcolmia*, which has been removed from the Anastaticeae, placed in its own tribe and has been reduced from 30 to six species (Al-Shehbaz 2014). Although the new tribal name Noccaeeae had been proposed to replace Coluteocarpeae (Al-Shehbaz et al. 2006), the old tribal name is still in use and is retained in the species checklist (Al-Shehbaz 2014).

The tribal placement of several unassigned genera listed in Al-Shehbaz (2012a) is the subject of on-going studies. In particular, these studies have resulted in the transfer of several genera between tribes, in the placement of previously unassigned genera and the reduction in the size of several tribes. Amongst the genera transferred, *Parodiodoxa* has been assigned to the Thelypodieae (Salariato et al. 2013b); *Heldreichia*, *Ricotia* and *Lunaria* have been assigned to the Biscutelleae (Özüdoğru et al. 2017); *Camelinopsis* has been replaced by *Pseudocamelina* and placed in the Thlaspideae (Esmailbegi et al. 2017) and *Pseudofortuynia* has been merged with *Sisymbrium* and placed in the Sisymbrieae (German and Al-Shehbaz 2018). The genus *Stenodraba* has been re-instated and its species have been moved from the Halimolobeae and Thelypodieae tribe to the Eudemeae (Salariato et al. 2018). The genus *Hilliella* has been transferred from the Yinshanieae to the new tribe Hillielleae (Chen et al. 2016, Al-Shehbaz 2017b). A recent wide-ranging study of the Brassicaceae (Nikolov et al. 2019) includes recommendations for tribal assignments of other unplaced taxa: that *Andrzeiowskia* be assigned to the Cardamineae, *Ochthodium* to the Sisymbrieae, *Petrocallis* to the Kernereae, *Raphanoryncha* to the Thelypodieae and *Veselskya* to the Anchonieae. The authors also recommended that new tribes be created to accommodate *Dipoma*, *Fourraea*, *Hemilophia* and *Schrenkiella*. The tribal placement of the remaining unassigned genera on the list, *Asperuginoides*, *Chamira* and *Idahoa*, has not been resolved.

Significant generic changes have occurred since the previous version of the checklist. The following 17 genera have been re-instated: *Abdra* (Jordon-Thaden et al. 2010), *Acuston* (Španiel et al. 2015), *Brachypus* (Španiel et al. 2015), *Drabella* (Jordon-Thaden et al. 2010), *Guenthera* (Warwick and Hall 2009), *Hilliella* (Chen et al. 2016), *Irania* (Španiel et al. 2015), *Lepidotrichum* (Španiel et al. 2015), *Lutzia* (Španiel et al. 2015), *Machaerophorus* (Salariato et al. 2019), *Meniocus* (Španiel et al. 2015), *Micrantha* (Al-Shehbaz 2012a), *Odontarrhena* (Španiel et al. 2015), *Parryodes* (Koch et al. 2012a), *Phyllolepidium* (Cecchi 2011), *Tomostima* (Al-Shehbaz et al. in Al-Shehbaz 2012a) and *Stenodraba* (Salariato et al. 2018). Additionally, a few *Guenthera* taxa, formerly subsumed in the synonymy of *Brassica*, have been restored as distinct from *Brassica* (German 2015). Twenty-one genera have been created: *Aimara* (Salariato et al. 2013a), *Alshehbazia* (Salariato and Zuloaga 2015), *Anzhengxia* (Al-Shehbaz and German 2016), *Atacama* (Toro-Núñez et al. 2015), *Bengt-jonsellia* (Al-Shehbaz 2017c), *Borodiniopsis* (Koch et al. 2012a), *Cuprella* (Salmeron-Sanchez et al. in Španiel et al. 2015), *Lysakia* (Esmailbegi & Al-Shehbaz in Esmailbegi et al. 2018), *Marcus-Kochia* (Al-Shehbaz in Al-Shehbaz et al. 2014), *Metashangrilaia* (Al-Shehbaz and German 2016), *Mummenhoffia* (Esmailbegi & Al-Shehbaz in Esmailbegi et al. 2018), *Pterygostemon* (Španiel et al. 2015), *Resetnikia* (Španiel et al. 2015), *Rudolf-kamelinia* (Al-Shehbaz and German 2016), *Scapiarabis* (Koch et al. 2012a), *Shehbazia* (German and Friesen 2014), *Sinalliaria* (Jin et al. in Zhou et al. 2014), *Sinoarabis* (Koch et al. 2012a), *Terraria* (Hildebrand and Al-Shehbaz 2017), *Yosemitea* (Alexander & Windham in Alexander et al. 2013) and *Zuloagocardamum* (Salariato and Al-Shehbaz 2014). Ten genera have been considerably expanded: *Borodinia* (Alexander & Windham in Alexander et al. 2013), *Braya* (Al-Shehbaz and German 2014), *Draba* (Al-Shehbaz 2007, Al-Shehbaz and Mulligan 2013), *Eutrema* (Al-Shehbaz et al. in Hao et al. 2017), *Ionopsidium* (Koch 2012), *Lepidium* (Al-Shehbaz 2010), *Mostacillastrum* (Al-Shehbaz 2012b), *Neuontobotrys* (Al-Shehbaz 2012a, Al-Shehbaz 2017a, Al-Shehbaz et al. 2013), *Noccaea* (Al-Shehbaz 2014) and *Parrya* (Al-Shehbaz and German 2013). Significant reductions have been made to *Alyssum* following the re-instatement of *Lutzia*, *Meniocus* and *Odontarrhena* (Španiel et al. 2015) and to *Thlaspi*, as a result of many transfers to *Noccaea* (Al-Shehbaz 2014). Finally, 36 genera have been subsumed into synonymy: *Acanthocardamum*, *Achoriphragma*, *Berteroella*, *Boreava*, *Brossardia*, *Callothlaspi*, *Camelinopsis*, *Catadysia*, *Caulanthus*, *Chalcanthus*, *Coelophragmus*, *Coluteocarpus*, *Elburzia*, *Eremodraba*, *Eunomia*, *Guillenia*, *Gynophorea*, *Hutchinsia*, *Microthlaspi*, *Moriera*, *Oreoblastus*, *Oreoloma*, *Petiniotia*, *Phaeonychium*, *Physocardamum*, *Pseudoclausia*, *Pseudofortuynia*, *Sameraria*, *Schivereckia*, S*ibaropsis*, *Spirorhynchus*, *Streptanthella*, *Tauscheria*, *Tchihatchewia*, *Transberingia* and *Vania*.

Recently, there has been a growing emphasis by botanists on the re-examination of herbarium specimens to establish which specimens clearly represent the holotype of an author’s species and which ones require a new designation. Occasional designations of a type by botanists go back over a century, but papers have been appearing in recent decades dedicated in part or even entirely to typification of species. Amongst such papers are Al-Shehbaz (2010), Al-Shehbaz (2015), Al-Shehbaz et al. (2011a), Al-Shehbaz in Cafferty and Jarvis (2002) and Al-Shehbaz in Moazzeni et al. (2018), German (2012), German (2014), German and Al-Shehbaz (2015), Marhold in Marhold et al. (2007), Marhold et al. (2015) and Salariato et al. (2014). Information on these efforts has been added to this version of the species checklist database. Additional recent publications used to build the checklist are included in Suppl. material 1.

Following the extensive taxonomic changes in the Brassicaceae, we provide an updated checklist that follows biodiversity data standards for publication, making this new version easily accessible and interoperable. Taxon names and authorities in the checklist were compared to the International Plant Names Index (IPNI) and BrassiBase, a Brassicaceae knowledge system maintained by experts in the plant family (Koch et al. 2012b, Kiefer et al. 2014, Koch et al. 2018).

## Project description

### Title

Update on the Brassicaceae Species Checklist

## Geographic coverage

### Description

The checklist covers species, subspecies and varieties from the Brassicaceae family at a global scale.

### Coordinates

-90 and 90 Latitude; -180 and 180 Longitude.

## Taxonomic coverage

### Description

The database includes information on 14983 taxa, 4636 of which are currently accepted and divided into 340 genera and 52 tribes.

### Taxa included

**Table:** 

Rank	Scientific Name	Common Name
kingdom	Plantae	plants
class	Equisetopsida	vascular plants
family	Brassicaceae	mustard or crucifer family

## Traits coverage

### Data coverage of traits

PLEASE FILL IN TRAIT INFORMATION HERE

## Temporal coverage

### Notes

The database includes taxa published from 1970 through to 2019.

## Usage licence

### Usage licence

Creative Commons Public Domain Waiver (CC-Zero)

### IP rights notes

This work is licensed under a Creative Commons Attribution (CC-BY) 4.0 License.

## Data resources

### Data package title

Update on the Brassicaceae Species Checklist

### Resource link

https://www.gbif.org/dataset/94308742-058c-46d5-b763-06e9207a6b15

### Alternative identifiers

http://ipt.pensoft.net/resource?r=aafc-brassicaceae-checklist

### Number of data sets

1

### Data set 1.

#### Data set name

Update on the Brassicaceae Species Checklist

#### Data format

Darwin Core

#### Number of columns

13

#### Description

**Data set 1.** 

Column label	Column description
id	Row number.
taxonID	A unique identifier for the set of nomenclatural and taxonomic information (data associated with the Taxon class).
acceptedNameUsageID	The taxonID of the taxon considered to be the accepted name for this nameUsage.
scientificName	The taxon name with authorship information, if applicable.
acceptedNameUsage	The scientificName of the taxon considered to be the accepted name for this nameUsage.
originalNameUsage	The equivalent of the scientificName as it originally appeared when the name was first established under the rules of the associated nomenclaturalCode (i.e. within the namePublishedIn reference). The basionym of the scientificName or the senior/earlier homonym for replaced names.
namePublishedIn	Reference to a publication representing the original publication of the name.
genus	The full scientific name of the genus in which the taxon is classified.
specificEpithet	The name of the species epithet of the scientificName.
infraspecificEpithet	The name of the lowest or terminal infraspecific epithet of the scientificName, excluding any rank marker.
taxonRank	The taxonomic rank of the most specific name in the scientificName.
taxonomicStatus	The status of the use of the scientificName as a label for a taxon.
taxonRemarks	Comments or notes about the taxon or name. Includes the tribe designation of the taxon.

## Additional information

### Updates from the previous version

The checklist presented here (Francis et al. 2020) was compiled from a careful evaluation of the taxonomic and molecular literature, comprising not only publications since 2006, but also previously unseen publications now available through various digitisation programmes. This update was the result of amendments to the original checklist, as well as the addition of both recently-published taxa and taxa not included in the scope of the original checklist and species in use within floras from around the world (Warwick et al. 2006), but which have been cited as synonyms or lectotypes in recent literature.

In total, 1212 new taxa were included, 131 taxa moved to a different genus, 1970 taxa changed status, the authority of 3672 taxa was changed to meet the well-established abbreviation guidelines (Brummitt and Powell 1992) and 685 authorities were updated. The additional fields on tribal affiliations and typification of herbarium specimens required further research in literature pertaining generally to the Brassicaceae family.

The fields in this new version of the checklist were mapped to the appropriate Darwin Core (DwC) terms to meet biodiversity data standards. This checklist was then published in the Pensoft Integrated Publishing Toolkit (IPT, http://ipt.pensoft.net/rss.do) and published in GBIF (https://doi.org/10.15468/rb7kky).

### Data validation

To validate the checklist, taxon names and their authority, including the basionym authority, were matched to IPNI using the Global Names Resolver (gnr_resolve) from the R package taxize (0.9.99). To compare with other available Brassicaceae resources, BrassiBase (v1.3) taxon names and their authority were also matched to IPNI, as described above. At the time of the analysis, gnr_resolve had last updated their IPNI database on 28-05-2020. The data and accompanying code were version-tracked using Git and are available in the public repository (https://bitbucket.org/bibilujan/global_brassicaceae_checklist/src/master/).

**Figure 1.**
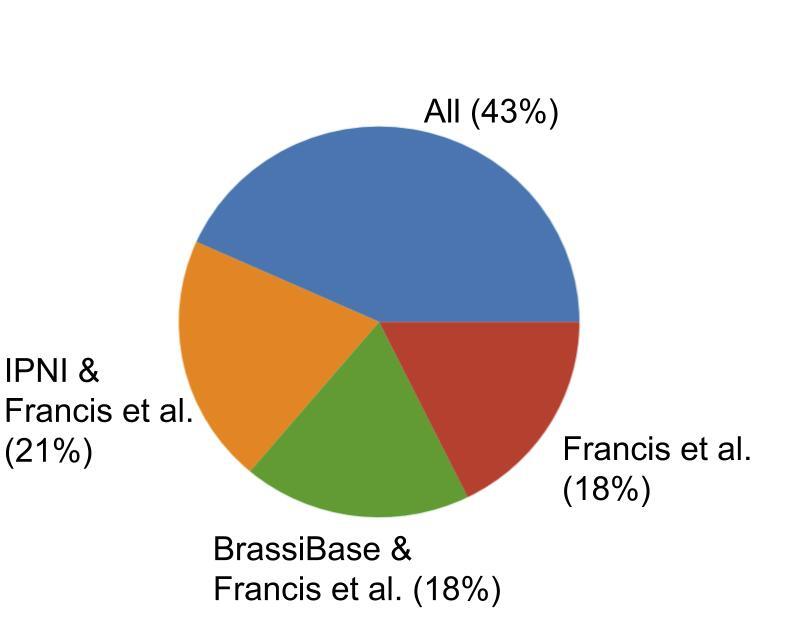
Proportion of taxa names with their authority from the checklist presented here (Francis et al.) that had an exact match to the International Plant Names Index (IPNI) and BrassiBase v1.3 (BrassiBase) and those that were common in all three resources (All).

Comparison to IPNI and BrassiBase showed a large overlap amongst the three resources. Forty-three percent of the names were consistent in all three resources and 18% were uniquely represented in the checklist presented here, with no exact match to IPNI or BrassiBase (Fig. 1).

Authority abbreviation format in the checklist presented here was more consistent with IPNI than BrassiBase; when whitespace is ignored, more of the BrassiBase names are consistent with IPNI and Francis et al. (Table 1).

From the total 14,983 names and their authority in the Brassicaceae Checklist, 64% were found to match IPNI (Table 2). From the remaining 36%, nearly a third of the names (1690, 11%) matched IPNI, but the authority was different and a similar number of names and their authority (1165, 8%) matched IPNI, but the basionym authority was missing in IPNI or did not match (Suppl. material 2). Finally, 335 names were found to have a different spelling when compared to IPNI (Suppl. material 3).

### Conclusions and future steps

The checklist, presented here, provides a significant update to the first version (Warwick et al. 2006). The previous version of the database was made available through a CD-ROM in 2006; since then, great advances have been made in science and technology that have greatly improved ease of sharing and publishing data. Despite those advances, there is still work needed to make these data open and shareable. Many resources still exist in silos with unique data fields, making data discoverability and interoperability difficult. Recently, a workflow has been published to facilitate the process of making species checklists more discoverable and easy to use for research and to support policy development (Reyserhove et al. 2020). Many of the recommendations by Reyserhove and collaborators had already been followed for the production of this new checklist (Francis et al. 2020).

Efforts have been made in this release of the Brassicaceae Checklist to meet current standards of data publishing: columns in the database have been converted to DwC terms, version control has been used and the checklist has been published through an IPT and GBIF. The names in the checklist were matched to IPNI and BrassiBase for comparison and a large overlap was found amongst the three resources (Fig. 1, Table 1). Comparison to IPNI showed discrepancies that need to be resolved in taxon authorities and the spelling of names (Suppl. materials 2, 3). Publishing the checklist through an IPT allows this resource to continue to evolve as new data become available. Future updates of this checklist can address the issues discovered here.

## Supplementary Material

BCEB0AD3-2432-5396-8A50-5F14362BFB11 10.3897/BDJ.9.e58773.suppl1 Supplementary material 1 Additional References Data type References Brief description Additional recent and relevant publications used to build the checklist. File: oo_453956.pdf https://binary.pensoft.net/file/453956 Francis A, Lujan-Toro BE, Warwick SI, Macklin JA, Martin SL

CF77E15F-DF4D-5B0D-9A10-7F2C33131BD6 10.3897/BDJ.9.e58773.suppl2 Supplementary material 2 Differences in taxon authority between the Francis et al. Brassicaceae checklist and IPNI Data type Table Brief description Taxon names and their taxonID from the Brassicaceae checklist that had a different authority when compared to the International Plant Names Index (IPNI) using the Global Names Resolver (gnr_resolve) from the R package taxize. File: oo_494033.csv https://binary.pensoft.net/file/494033 Francis A, Lujan-Toro BE, Warwick SI, Macklin JA, Martin SL

7F6DBEE0-5A45-5BD6-B46F-C6EC1755678A 10.3897/BDJ.9.e58773.suppl3 Supplementary material 3 Differences in scientific names and their taxonID between the Francis et al. Brassicaceae checklist and IPNI Data type Table Brief description Scientific names of taxa in the Brassicaceae checklist that had a different spelling than their match in the International Plant Names Index (IPNI) when compared using the Global Names Resolver (gnr_resolve) from the R package taxize. File: oo_494034.csv https://binary.pensoft.net/file/494034 Francis A, Lujan-Toro BE, Warwick SI, Macklin JA, Martin SL

## Figures and Tables

**Table 1.** Proportion and total number of taxon names and their authority from the Brassicaceae checklist (Francis et al.) that were shared with IPNI and BrassiBase, after performing an exact match and a match that ignores spaces.

**Match Type**	**Exact match**		**Ignore spaces**	
	**Count**	**Percentage (%)**	**Count**	**Percentage (%)**
All	6485	43	9076	60
Francis et al. & IPNI	3083	21	532	4
Francis et al. & BrassiBase	2762	18	3984	27
Francis et al.	2653	18	1391	9

**Table 2.** Proportion and total number of taxon names and their authority from the Brassicaceae checklist after matching to IPNI using Global Names Resolver from the R package taxize.

**Match to IPNI**	**Count**	**Percentage (%)**
Exact	9568	64
No match to IPNI	2225	15
Author no match	1690	11
Basionym author no match	1165	8
Name spelling no match	335	2
