# Supplementary material for: Update on the Brassicaceae species checklist
Source: Biodivers Data J. 2021 Mar 4;9:e58773. doi: 10.3897/BDJ.9.e58773 (PMC7952366; doi:10.3897/BDJ.9.e58773)
Supplement: Supplementary material 1 — Additional References [file bdj-09-e58773-s001.pdf]

Additional References:

Alexander PJ, Windham MD, Govindarajulu R, Al-Shehbaz IA, Baley CD (2010) Molecular Phylogenetics and Taxonomy of the Genus *Thysanocarpus* (Brassicaceae). Systematic Botany 35 (3): 559‑577. https://doi.org/10.1600/036364410792495926

Al-Shehbaz IA (2006) The genus Sisymbrium in South America, with synopses of the genera *Chilocardamum*, *Mostacillastrum*, *Neuontobotrys*, and *Polypsecadium* (Brassicaceae). Darwiniana 44: 341‑358. http://www.ojs.darwin.edu.ar/index.php/darwiniana/article/view/110/99

Al-Shehbaz IA, Mutlu B, Dönmez AA (2007) The Brassicaceae (Cruciferae) of Turkey, Updated. Turkish Journal of Botany 31: 327‑336. https://journals.tubitak.gov.tr/botany/abstract.htm?id=9033

Al-Shehbaz IA (2009) Two New North American Species of Draba (Brassicaceae): *D. Heilii* from New Mexico and *D. Mulliganii* from Alaska. Harvard Papers in Botany 14(1): 83-86. https://doi.org/10.3100/025.014.0112

Al-Shehbaz IA, Windham MD (2009) New and noteworthy North American *Draba* (Brassicaceae). Harvard Papers in Botany 12:409‑419. https://doi.org/https://doi.org/10.3100/1043-4534(2007)12[409:nonnad]2.0.co;2

Al-Shehbaz IA (2010) A Synopsis of the Genus *Sibara* (Brassicaceae). Harvard Papers in Botany 15 (1): 139‑147. https://doi.org/10.3100/025.015.0107

Al-Shehbaz IA, Mummenhoff K (2011) *Stubendorffia* and *Winklera* belong to the expanded *Lepidium* (Brassicaceae). Edinburgh Journal of Botany 68:165‑171. https://doi.org/https://doi.org/10.1017/s0960428611000060

Al-Shehbaz IA (2012) A Synopsis of the South American *Descurainia* and Description of the Colombian New Species *D. cleefii* (Brassicaceae). Harvard Papers in Botany 17 (2): 221‑229. https://doi.org/10.3100/025.017.0202

Al-Shehbaz IA (2013a) A Revision of the Central American Genus *Romanschulzia* (Brassicaceae). Harvard Papers in Botany 18(1): 1‑12. https://doi.org/10.3100/025.018.0102

Al-Shehbaz IA (2013b) Clypeola is United with *Alyssum* (Brassicaceae). Harvard Papers in Botany 18(2): 125‑128. https://doi.org/10.3100/025.018.0204

Al-Shehbaz IA, Mayer MS (2013) New or Noteworthy *Streptanthus* (Brassicaceae) for the Flora of North America. Novon 18: 279‑282. https://doi.org/10.3417/2007163

Al-Shehbaz IA, Cano A, Manchego MAC, Salariato DL (2018) Remarkable discoveries in the long-neglected and peruvian-endemic genus *Machaerophorus* (Brassicaceae). Phytotaxa 360 (2):114‑124. https://doi.org/10.11646/phytotaxa.360.2.3

Bailey CD, Al-Shehbaz IA, Rajanikanth G (2007) Generic limits in tribe Halimolobeae and description of the new genus *Exhalimolobos* (Brassicaceae). Systematic Botany 32: 140–156. https://doi.org/10.1600/036364407780360166

Çetin Ö, Duran A, Martin E, Tuştaş S (2012) A taxonomic study of the genus *Fibigia* Medik. (Brassicaceae). Afr. J. Biotechnol. 11; 109–119. https://www.ajol.info/index.php/ajb/article/view/93046

de Lange PJ, Heenan PB, Houliston GJ, Rolfe JR, Mitchell AD (2013) New *Lepidium* (Brassicaceae) from New Zealand. Phytokeys 24: 1–147. https://doi.org/10.3897/phytokeys.24.4375

Esmailbegi S, Lysak MA, Rahiminejad MR, Mirtadzadini M, Mummernhoff K, Al-Shehbaz IA (2017b) A taxonomic revision of the genus *Graellsia* (Brassicaceae, tribe Thlaspideae). Phytotaxa 313(1): 105‑116. https://doi.org/10.11646/phytotaxa.313.1.7

German DA, Al-Shehbaz IA (2008) *Dendroarabis*, A Newasian Genus of Brassicaceae. Harvard Papers in Botany 13 (2): 289‑291. https://doi.org/10.3100/1043-4534-13.2.289

German DA (2013) Proposal to conserve the name *Alyssum canescens* (*Ptilotrichum canescens*, *Stevenia canescens*) (Cruciferae) with a conserved type. Taxon 62: 836‑837. https://doi.org/https://doi.org/10.12705/624.18

German DA (2014) New synonyms and combinations in Eurasian Brassicaceae (Cruciferae). Phytotaxa 173 (1): 31‑40. https://doi.org/10.11646/phytotaxa.173.1.2

German DA, Al-Shehbaz IA (2015) Proposal to conserve the name *Sisymbrium pumilum* Stephan, non Lam. (*Calymmatium pumilum*, *Olimarabidopsis pumila*, *Thellungiella pumila*) (Cruciferae). Taxon 64 (4): 849‑850. https://doi.org/10.12705/644.21

German DA (2016) Taxonomic notes on miscellaneous Cruciferae. Turczaninowia 19: 129‑135. https://doi.org/https://doi.org/10.14258/turczaninowia.19.4.17

German DA, Tekin M, Španiel S, Marhold K, Al-Shehbaz IA (2016) A brief taxonomic revision of *Physoptychis* (Alysseae, Brassicaceae). Phytotaxa 258 (1): 75‑82. https://doi.org/10.11646/phytotaxa.258.1.5

Hall JC, Tisdale TE, Donohue K, Wheeler A, Al-Yahya MA, Kramer EM (2011) Convergent evolution of a complex fruit structure in the tribe Brassiceae (Brassicaceae). American Journal of Botany 98 (12): 1989‑2003. https://doi.org/10.3732/ajb.1100203

Heenan PB, Goeke DF, Houliston GJ, Lysak MA (2012) Phylogenetic analyses of ITS and rbcL DNA sequences for sixteen genera of Australian and New Zealand Brassicaceae result in the expansion of the tribe Microlepidieae. Taxon 61 (5): 970‑979. https://doi.org/10.1002/tax.615004

Heenan PB (2017) A taxonomic revision of *Cardamine* L. (brassicaceae) in New Zealand. Phytotaxa 330 (1): 1‑154. https://doi.org/10.11646/phytotaxa.330.1.1

Hu H, Al-Shehbaz IA, Sun Y, Hao G, Wang Q, Liu J (2015) Species delimitation in *Orychophragmus* (Brassicaceae) based on chloroplast and nuclear DNA barcodes. Taxon 64 (4): 714-726. https://doi.org/10.12705/644.4

Koch MA, Al-Shehbaz IA (2009). Molecular systematics and evolution: Recognition of infrafamiliar taxa: the tribal system. In Gupta SK Biology and breeding of crucifers. CRC Press.

Mandáková T, Mummernhoff K, Al-Shehbaz IA, Mucina L, Mühlhausen A, Lysak MA (2012) Whole-genome triplication and species radiation in the southern African tribe Heliophileae (Brassicaceae). Taxon 61:989-1000. https://doi.org/https://doi.org/10.1002/tax.615006

Marhold K (2011) Brassicaceae. In: Euro+Med Plantbase - the information resource for Euro-Mediterranean plant diversity. http://ww2.bgbm.org/EuroPlusMed/. Accessed on: 2016-11-14.

Marhold K, Ŝlenker M, Kudoh H, Zozomová-Lihová J (2016) *Cardamine occulta*, the correct species name for invasive Asian plants previously classified as *C. flexuosa*, and its occurrence in Europe. PhytoKeys 62(1): 57-72. https://doi.org/10.3897/phytokeys.62.7865

Parolly G, Nordt B, Bleeker W, Mummenhoff K (2010) *Heldreichia* Boiss. (Brassicaceae) revisited: A morphological and molecular study. Taxon 59 (1): 187-202. https://doi.org/10.1002/tax.591018

Prina AO, Martínez-Laborde JB (2008) A taxonomic review of *Crambe*, sect. Dendrocrambe (Brassicaceae). Botanical Journal of the Linnean Society. 156: 291–304. https://doi.org/10.1111/j.1095-8339.2007.00708.x

Prina, AO (2009) Taxonomic review of the genus *Crambe*, sect. Crambe (Brassicaceae, Brassiceae). Anal. Jard. Bot. Madrid 66: 7–24. https://doi.org/10.3989/ajbm.2186

Ranjbar M, Karami S, Rostami M (2008) A revision of *Fibigia* sect. Purpureae (Brassicaceae, Alysseae) in Iran, and the description of three new species. Biological Diversity and Conservation 7 (1):32-43. https://doi.org/10.11646/phytotaxa.258.1.5

Salariato DL, Zuloaga FO, Al-Shehbaz IA. (2015a) A taxonomic revision of the genus *Xerodraba* (Eudemeae, Brassicaceae). Phytotaxa 207 (1): 39-67. https://doi.org/10.11646/phytotaxa.207.1.2

Salariato DL, Zuloaga FO, Cano A, Al-Shehbaz IA (2015b) Molecular phylogenetics of tribe Eudemeae (Brassicaceae) and implications for its morphology and distribution. Molecular Phylogenetics and Evolution 82 (PA): 43-59. https://doi.org/10.1016/j.ympev.2014.09.030

Salariato DL, Zuloaga FO, Frankze A, Mummenhoff K, Al-Shehbaz IA (2016). Diversification patterns in the CES clade (Brassicaceae tribes Cremolobeae, Eudemeae, Schizopetaleae) in Andean South America. Botanical Journal of the Linnean Society 181(4): 543-566. https://doi.org/10.1111/boj.12430

Warwick SI, Francis A, Al-Shehbaz IA (2006) Brassicaceae: Species checklist and database on CD-Rom. Pl. Syst. Evol. 259: 249-258. https://doi.org/10.1007/s00606-006-0422-0

Windham MD, Al-Shehbaz IA (2006) New and noteworthy species of *Boechera* (Brassicaceae) I: sexual diploids. Harvard Papers in Botany 11(1): 61‑88. https://doi.org/10.3100/1043-4534(2006)11[61:NANSOB]2.0.CO;2

Windham MD (2007a) New and noteworthy species of *Boechera* (Brassicaceae) II: Apomictic hybrids. Harvard Papers in Botany 11: 257‑274. https://doi.org/10.3100/1043-4534(2007)11[257:NANSOB]2.0.CO;2

Windham MD (2007b) New and noteworthy species of *Boechera* (Brassicaceae) III: Additional sexual diploids and apomictic hybrids. Harvard Papers in Botany 12: 235‑257. https://doi.org/10.3100/1043-4534(2007)12[235:NANSOB]2.0.CO;2

Yue J, Sun H, Li J, Al-Shehbaz IA (2008) A Synopsis Of An Expanded *Solms-laubachia* (Brassicaceae), and the Description Of Four New Species From Western China 1. Annals of the Missouri Botanical Garden 95 (3): 520‑538. https://doi.org/10.3417/2006214

Zozomová-Lihová J, Marhold K, Španiel S (2014) Taxonomy and evolutionary history of *Alyssum montanum* (Brassicaceae) and related taxa in southwestern Europe and Morocco: Diversification driven by polyploidy, geographic and ecological isolation. Taxon 63(3): 562‑591. https://doi.org/10.12705/633.18
